# A Treatise on Practical Pharmacy

**Published:** 1864-03

**Authors:** 


For sale by W. B. Keen & Co., Lake street, Chicago.

---

A Treatise on Practical Pharmacy. Designed as a Text-Book for the Student, and as a Guide for the Physician and Pharmaceutist. Containing the Official and many Unofficial Formulas, and numerous examples of Extemporaneous Prescriptions. By Edward Parrish, Graduate in Pharmacy; Member of the Philadelphia College of Pharmacy; of the Academy of Natural Sciences of Philadelphia; and of the American Pharmaceutical Association; Principal of the School of Practical Pharmacy of Philadelphia. Third Edition. Thoroughly Revised and Improved, with Important Additions. With Two Hundred and Thirty-eight Illustrations. Philadelphia: Blanchard & Lea. 1864.

This is a large-sized octavo volume of 850 pages. The former editions have been sufficiently long before the medical public to render the merits of the work well known. It is certainly one of the most complete and valuable works on practical pharmacy to which the student, the practitioner, or the apothecary can have access.

